# VAPB/ALS8 interacts with FFAT-like proteins including the p97 cofactor FAF1 and the ASNA1 ATPase

**DOI:** 10.1186/1741-7007-12-39

**Published:** 2014-05-29

**Authors:** Yorann Baron, Patrick G Pedrioli, Kshitiz Tyagi, Clare Johnson, Nicola T Wood, Daniel Fountaine, Melanie Wightman, Gabriela Alexandru

**Affiliations:** 1 Medical Research Council Protein Phosphorylation and Ubiquitylation Unit (MRC-PPU), College of Life Sciences, University of Dundee, Dow St, Dundee DD1 5EH, UK

**Keywords:** p97 ATPase, VAPB, FAF1, ASNA1, RPN2, ubiquitin

## Abstract

**Background:**

FAF1 is a ubiquitin-binding adaptor for the p97 ATPase and belongs to the UBA-UBX family of p97 cofactors. p97 converts the energy derived from ATP hydrolysis into conformational changes of the p97 hexamer, which allows the dissociation of its targets from cellular structures or from larger protein complexes to facilitate their ubiquitin-dependent degradation. VAPB and the related protein VAPA form homo- and heterodimers that are anchored in the endoplasmic reticulum membrane and can interact with protein partners carrying a FFAT motif. Mutations in either VAPB or p97 can cause amyotrophic lateral sclerosis, a neurodegenerative disorder that affects upper and lower motor neurons.

**Results:**

We show that FAF1 contains a non-canonical FFAT motif that allows it to interact directly with the MSP domain of VAPB and, thereby, to mediate VAPB interaction with p97. This finding establishes a link between two proteins that can cause amyotrophic lateral sclerosis when mutated, VAPB/ALS8 and p97/ALS14. Subsequently, we identified a similar FFAT-like motif in the ASNA1 subunit of the transmembrane-domain recognition complex (TRC), which in turn mediates ASNA1 interaction with the MSP domain of VAPB.

Proteasome inhibition leads to the accumulation of ubiquitinated species in VAPB immunoprecipitates and this correlates with an increase in FAF1 and p97 binding. We found that VAPB interaction with ubiquitinated proteins is strongly reduced in cells treated with FAF1 siRNA. Our efforts to determine the identity of the ubiquitinated targets common to VAPB and FAF1 led to the identification of RPN2, a subunit of an oligosaccharyl-transferase located at the endoplasmic reticulum, which may be regulated by ubiquitin-mediated degradation.

**Conclusions:**

The FFAT-like motifs we identified in FAF1 and ASNA1 demonstrate that sequences containing a single phenylalanine residue with the consensus (D/E)(D/E)FEDAx(D/E) are also proficient to mediate interaction with VAPB.

Our findings indicate that the repertoire of VAPB interactors is more diverse than previously anticipated and link VAPB to the function of ATPase complexes such as p97/FAF1 and ASNA1/TRC.

## Background

FAF1 (also known as UBXN3A or UBXD12) is a p97 cofactor from the UBX-domain family. Humans express 13 UBX-domain proteins, most of which interact with the p97 ATPase via their C-terminal UBX-domain [1,2]. Exceptionally, UBXD1 does not interact with the p97 N-terminus via its UBX domain, but uses its PUB domain to bind the C-terminus of p97 [3,4]. Five of these cofactors, including FAF1, have been termed UBA-UBX proteins due to the presence of a ubiquitin-associated (UBA) domain at their N-terminus, which mediates interaction with ubiquitinated proteins. The general function of UBA-UBX proteins is that of ubiquitin-binding adaptors for the p97 ATPase [1,5-8]. Furthermore, three UBA-UBX proteins – UBXD7 (or UBXN7), UBXD8 (also known as UBXN3B, FAF2 or ETEA) and FAF1 – all have a central UAS domain, which was recently shown to interact with long-chain unsaturated fatty acids thereby mediating UBA-UBX protein oligomerization [9]. However, the various UBA-UBX proteins are not functionally redundant, due, at least in part, to the presence of specific domains that are found in a single UBA-UBX protein. For example, the UIM domain, which is only present in UBXD7, allows this protein to interact specifically with the NEDD8 modification on cullins [10,11]. Owing to their ATPase activity, p97 complexes function as ‘segregases’, which can dissociate their targets from protein partners [12,13] or even retrotranslocate proteins from the endoplasmic reticulum (ER) back into the cytosol to allow for their ubiquitination and degradation [14,15]. This latter function is fundamental for p97’s role in ER-associated degradation (ERAD), a quality control pathway that ensures degradation of ER proteins that are misfolded or misassembled [16].

Vesicle-associated proteins (VAP) are highly conserved across eukaryotes, from yeast to mammals [17-23]. Humans express two VAP proteins, VAPA and VAPB, whose primary sequence is 63% identical. Both are membrane-anchored at the ER and Golgi via C-terminal transmembrane domains [20,22] and can exist as homo- or heterodimers [18]. Oligomerization is largely mediated by a cytoplasmic coiled-coil region, with some contribution from the transmembrane domain [24,25]. VAP proteins contain an MSP domain in their N-terminal half, which has been shown to interact with proteins containing a FFAT motif (two phenylalanines in an acidic tract motif), such as members of the oxysterol-binding protein (OSBP) family or the phosphatidylinositol transfer proteins from the PITPNM family [26]. The consensus sequence for the FFAT motif is EFFDAxE, with an acidic tract immediately upstream of it [27]. The crystal structure of the MSP domain bound to a FFAT peptide suggests that a VAP dimer may bind two FFAT motifs, with the FFATs engaging each other and both MSP domains of the dimer [28].

A Phe56 to Ser mutation in the MSP domain of VAPB causes amyotrophic lateral sclerosis (ALS) type 8 in humans, transmitted in an autosomally dominant manner [29]. Subsequently, another VAPB mutation in the same region (Thr46 to Ile) was identified in a British ALS patient [30]. ALS is a progressive neurodegenerative disorder, which affects upper and lower motor neurons and is lethal within 5 years of clinical onset [31]. Interestingly, in some cases mutations in p97 can also cause ALS [32,33].

Our mass spectrometry analysis of Flag-FAF1 immunoprecipitates led to the identification of VAPA and VAPB as potential interaction partners for FAF1. Given the implication of both VAPB and p97 in ALS, we decided to follow up on this interaction, aiming to understand whether FAF1 might link VAPB and p97 in a common functional pathway. We show that VAPB interaction with FAF1 is not mediated by ubiquitin-modification of VAPB, but it is due to the presence of a FFAT-like motif in FAF1. Further work identified a very similar FFAT-like motif in ASNA1, which we demonstrate is another novel binding partner for VAPB. Although VAPB itself is not a ubiquitinated target for FAF1/p97, we found that VAPB does interact with ubiquitinated proteins in a FAF1-dependent manner and, subsequently, identified RPN2 as a common ubiquitinated target for VAPB and FAF1.

## Results

### FAF1 interacts with VAPA and VAPB

**Figure 1:**
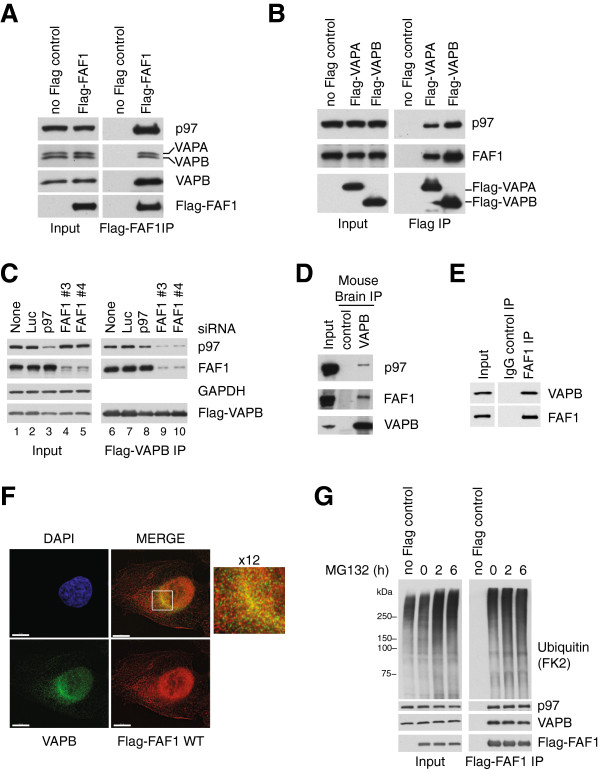
**FAF1 interacts with VAPA and VAPB. (A)** Flag-FAF1 immunoprecipitated from U2OS cells interacts with p97, VAPA and VAPB. **(B)** Flag-VAPA/B immunoprecipitated from U2OS cells interact with p97 and FAF1. **(C)** VAPB interaction with p97 is dependent on FAF1. U2OS cells were treated with the indicated siRNA oligos; luciferase (Luc) siRNA was used as a control. Flag-VAPB was immunoprecipitated and immunoblots of the immunoprecipitates (right) show that FAF1 depletion reduces the interaction with p97 whereas p97 depletion does not significantly affect the interaction with FAF1. **(D)** Endogenous VAPB interacts with p97 and FAF1 in mouse brain. Endogenous VAPB was immunoprecipitated from mouse brain extracts using Protein A-Sepharose (PAS) beads cross-linked to anti-VAPB antibodies. Uncoupled beads were used as a control. **(E)** Endogenous FAF1 interacts with VAPB in U2OS cells. The immunoprecipitation was performed using sheep anti-FAF1 antibody or sheep immunoglobulin G (IgG) as a control and PAS beads. **(F)** Indirect immunofluorescence of VAPB and wild-type (WT) Flag-FAF1. U2OS cells expressing Flag-FAF1 from a tetracycline-inducible promoter were grown in the presence of 200 ng/ml tetracycline for 24 hr and treated with 10 μM MG132 for 2 hr. Flag-FAF1 WT (red) co-localizes with VAPB (green) in a peri-nuclear area (enlarged window), suggesting an ER pattern. Scale bar is 10 μm. **(G)** VAPB levels and its interaction with Flag-FAF1 are not affected upon proteasome inhibition. Flag-FAF1 was immunoprecipitated from U2OS cells treated with 10 μM MG132 for 2 hr, 5 μM MG132 for 6 hr or left untreated (0 hr). Ubiquitinated proteins, p97 and VAPB were detected by immunoblotting in inputs (left) and immunoprecipitates (right). DAPI, 4',6-diamidino-2-phenylindole; IgG, immunoglobulin G; IP, immunoprecipitate; Luc, luciferase; WT, wild type.

Both VAPA and VAPB were identified by mass spectrometry in Flag-FAF1 immunoprecipitates from human U2OS cells (Table 1, Additional file 1: Table S1). We first sought to confirm these interactions by Western blotting. Indeed, VAPA and VAPB were detected in Flag-FAF1 immunoprecipitates from U2OS cells, using specific antibodies (Figure 1A). Conversely, both FAF1 and p97 were present in Flag-VAPA and Flag-VAPB immunoprecipitates from U2OS cells (Figure 1B). Next, we showed that Flag-VAPB interaction with p97 is drastically reduced in cells treated with FAF1 siRNA (Figure 1C, compare lanes 9 and 10 with 6 and 7). In contrast, treatment with p97 siRNA had little, if any, effect on FAF1 binding to Flag-VAPB (Figure 1C, lane 8). We therefore conclude that p97 binding to VAPB is mediated by FAF1.To verify that FAF1 and VAPB also interact at endogenous levels, we raised rabbit antibodies to VAPB and performed an immunoprecipitation from mouse brain extracts. As shown in Figure 1D, endogenous VAPB interacts with FAF1 and p97 in the brain. Reciprocally, endogenous FAF1 co-immunoprecipitated VAPB from U2OS cells (Figure 1E). Furthermore, we showed using immunofluorescence microscopy that both VAPB and Flag-FAF1 exhibit a punctate cytoplasmic staining and they are co-localized, most notably in the peri-nuclear region in a pattern typical for ER proteins (Figure 1F).

**Table 1:** VAPA and VAPB were identified by mass spectrometry in Flag-FAF1 immunoprecipitates

**Protein name**	**UniProt ID**	**MW (Da)**	**Share of spectrum IDs**^**a**^	**Sequence coverage**
VAPA	Q9P0L0	27,893	0.62%	63.9%
VAPB	O95292	27,228	0.75%	65.0%

^a^The share of spectrum IDs is indicated as a measure of protein abundance in the immunoprecipitates. Its value represents the percentage of peptide spectra assigned to VAPA/B out of all identified spectra. MW, molecular weight.

### VAPB is not targeted for ubiquitin-mediated degradation

Because FAF1 is a ubiquitin-binding adaptor for the p97 ATPase [1], we initially assumed that VAPB might be targeted for proteasomal degradation and interact with FAF1 in its ubiquitinated form. However, we found that proteasome inhibition with MG132 for 2 or 6 hr had no effect on VAPB levels (Figure 1G, left panel), nor could we detect ubiquitinated forms of VAPB even after a long exposure of Flag-VAPB immunoprecipitates (Additional file 2: Figure S1). Moreover, VAPB interaction with Flag-FAF1 was not affected by MG132 treatment although we could detect an increased binding of ubiquitinated proteins to FAF1 (Figure 1G, right panel). These data suggested that VAPB is not targeted for ubiquitin-mediated proteasomal degradation. Hence, VAPB interaction with FAF1 and p97 appears to serve a function other than facilitating VAPB degradation.

### FAF1 interaction with VAPB is mediated by a FFAT-like sequence in FAF1 and the MSP domain of VAPB

**Figure 2:**
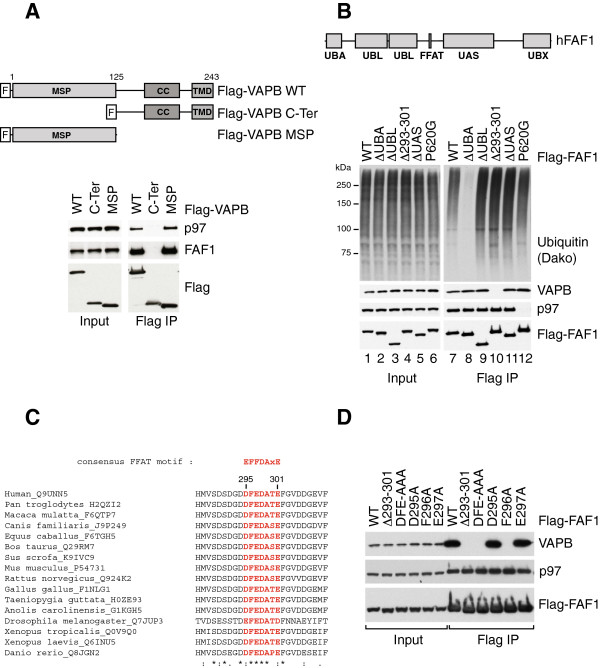
**A FFAT-like motif mediates FAF1 interaction with VAPB. (A)** The MSP domain of VAPB mediates its interaction with FAF1. Top panel: Schematic representation of the Flag-VAPB constructs, full length (1 to 243) or truncated, C-terminal half (125 to 243) and the MSP domain N-terminal half (1 to 125), immunoprecipitated from U2OS cells as shown in the bottom panel. Immunoblots of inputs (left) and immunoprecipitates (right) show that the MSP of VAPB is sufficient for the interaction with FAF1/p97. A highly conserved FFAT-like motif in FAF1 mediates its interaction with VAPB. **(B)** Top panel: Schematic representation of human FAF1 highlighting its various domains. Bottom panel: Wild-type or mutant variants of Flag-FAF1 were immunoprecipitated from U2OS cells. The indicated proteins were detected using specific antibodies in the inputs (left) and immunoprecipitates (right). UBA deletion caused a dramatic reduction in ubiquitinated protein binding to FAF1 whereas a point mutation in the UBX domain (P620G) abolished p97 binding. Deletion of the residues 293 to 301 was the only truncation that prevented VAPB binding to FAF1. **(C)** Alignment of FAF1 sequence from various species showing that the FFAT-like motif is highly conserved. **(D)** As in **(B)**, showing that either a triple mutation D295A/F296A/E297A or single F296A mutation in FAF1 abolished VAPB binding. IP, immunoprecipitate; WT, wild type.

To better understand the interaction of VAPB with FAF1, we tried to map the interaction domains in both proteins. Our task was very simple as far as VAPB is concerned. The N-terminal half of VAPB comprises an MSP domain, followed by a coiled-coil region and the transmembrane region at the extreme C-terminus. Upon over-expression in human cells, we found that the MSP domain alone was as competent in interacting with FAF1 and p97 as full-length VAPB (Figure 2A, right panel). In contrast, the C-terminal region of VAPB did not interact with FAF1/p97 at all.Mapping the interaction region in FAF1 turned out to be more challenging. The domain architecture of FAF1 comprises a UBA domain at the N-terminus, which mediates the interaction with ubiquitin, followed by two ubiquitin-like (UBL) domains, a UAS domain and the UBX domain at the C-terminus, which mediates interaction with p97 (Figure 2B, top panel). We expressed in human cells N-terminally Flag-tagged truncated versions of FAF1 lacking the UBA domain, the two UBLs, or the UAS and also FAF1 carrying a point mutation in the UBX domain (Pro620 to Gly). As expected, UBA deletion abolished ubiquitin binding and the P620G mutation abolished p97 binding. However, to our surprise, all these FAF1 variants had wild-type ability to interact with VAPB (Figure 2B, right panel).

Because the MSP domain of VAPB is known to interact with various proteins carrying a FFAT motif [26], we searched the FAF1 sequence for the presence of such a motif. There was no typical FFAT motif in FAF1. Yet in the region between the two UBLs and the UAS domain, we identified a short sequence that highly resembled FFAT motifs, the main difference being that in FAF1 the second phenylalanine residue is replaced by an additional acidic residue (Figure 2C). We therefore tested whether deletion of the FFAT-like region in FAF1 has any effect on VAPB binding. Indeed, the removal of only nine amino acids from residue 293 to 301 resulted in a complete loss of VAPB binding, while p97 and ubiquitinated protein binding remained unaffected (Figure 2B, lane 10). To exclude the possibility that this short truncation might affect FAF1 folding, we went on to create a triple mutant (DFE to AAA) and three individual point mutants in that region – D295A, F296A and E297A. Both the triple mutant and the F296A mutation abolished VAPB binding, while the other two point mutants retained the wild-type ability to interact with VAPB (Figure 2D). We therefore conclude that FAF1 interacts with VAPB via a highly conserved FFAT-like motif (Figure 2C), with the single phenylalanine residue present in this region being particularly important for the interaction.

### VAPB directly interacts with FAF1 *in vitro* and the interaction is not affected by the mutation causing amyotrophic lateral sclerosis

**Figure 3:**
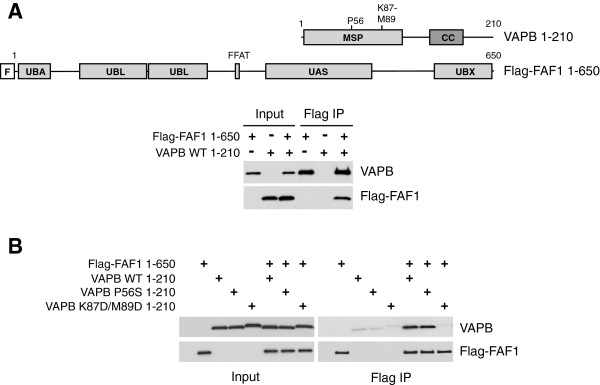
**The P56S ALS-causing mutation of VAPB does not affect its interaction with FAF1 *in vitro*.** The indicated variants of recombinant VAPB and Flag-FAF1 were incubated *in vitro* either alone or in combination, and then immunoprecipitated using anti-Flag beads. **(A)** Flag-FAF1 interacts directly with VAPB. Top panel: Schematic representation of the truncated version of VAPB (residues 1 to 210), lacking the C-terminal transmembrane region, and Flag-FAF1 full-length proteins expressed in bacteria. Bottom panel: Immunoblots of the indicated proteins in input extracts and anti-Flag immunoprecipitates. **(B)** The wild-type and the P56S mutant of VAPB (1 to 210), but not the K87D M89D double mutant, interact with Flag-FAF1 (right panel). IP, immunoprecipitate; WT, wild type.

Next, we checked whether recombinant VAPB and FAF1 could interact when mixed together *in vitro*. We expressed in bacteria full-length Flag-tagged FAF1 and a truncated version of VAPB lacking the C-terminal transmembrane region. Using anti-Flag beads, we were able to isolate both Flag-FAF1 and VAPB (Figure 3A), suggesting that VAPB interacts directly with FAF1 and does not require additional cofactors.

The P56S mutation in VAPB that was identified in ALS patients causes the protein to aggregate and to become insoluble when expressed in mammalian cells in culture [34]. Due to the difficulty of extracting VAPB P56S from human cells, it was not possible for us to study how this mutation might affect FAF1 binding in human cells. However, we found that both wild-type and VAPB P56S can be readily expressed in bacteria. Recombinant VAPB P56S retained the wild-type ability to interact with Flag-FAF1 *in vitro* (Figure 3B). In contrast, a VAPB double mutant known to be defective in FFAT binding, K87D M89D [28], could not be co-immunoprecipitated with FAF1 under the same conditions. These results indicate that the P56S mutation as such does not perturb VAPB interaction with FAF1.

### VAPB interaction with FAF1 and p97 is stimulated upon proteasome inhibition

**Figure 4:**
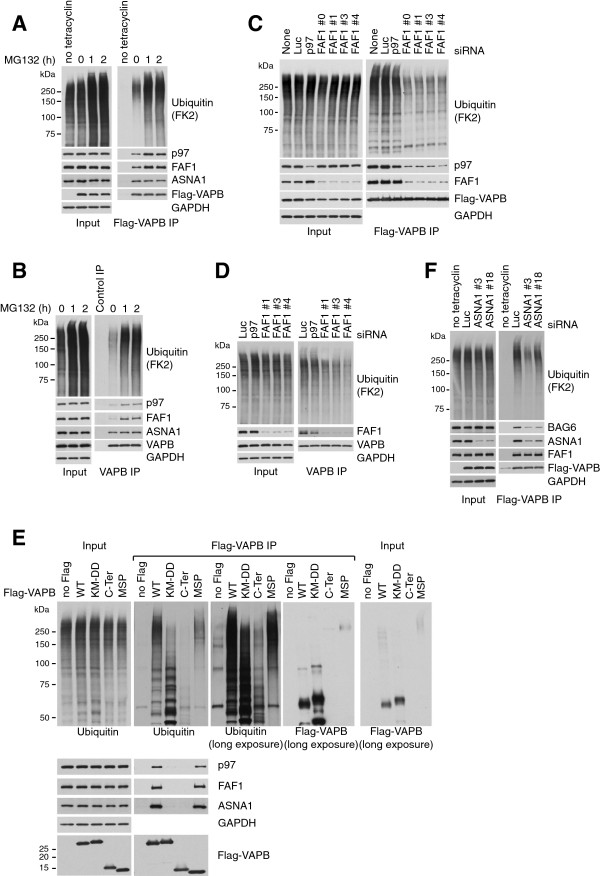
**VAPB interaction with FAF1 and p97 is stimulated upon proteasome inhibition. (A)** Proteasome inhibition enhances ubiquitin, p97 and FAF1 binding to Flag-VAPB, while ASNA1 binding remains largely unchanged. U2OS cells expressing Flag-VAPB from a tetracycline-inducible promoter were grown in the presence of 100 ng/ml tetracycline for 24 hr or left untreated as a control. Flag-VAPB was immunoprecipitated using anti-Flag beads. **(B)** Proteasome inhibition enhances ubiquitin, p97 and FAF1 binding to endogenous VAPB, while ASNA1 binding was only slightly increased. Endogenous VAPB was immunoprecipitated from U2OS cells using anti-VAPB antibodies cross-linked to Protein A-beads. Uncoupled beads were used as control. **(C, D)** The binding of ubiquitinated proteins to VAPB is largely mediated by FAF1. U2OS cells were treated with the indicated siRNA oligos. Flag-VAPB **(C)** or endogenous VAPB **(D)** were immunoprecipitated as described above. Depletion of FAF1 strongly reduces the binding of ubiquitinated proteins to Flag- or endogenous VAPB. **(E)** The K87D M89D double mutant (KM-DD) of VAPB is defective in p97, FAF1, ASNA1 and ubiquitin binding. Flag-VAPB full-length WT, KM-DD and truncated, C-terminal half (C-Ter) and N-terminal half (MSP), were immunoprecipitated from U2OS cells. The MSP domain of VAPB is sufficient to interact with p97, FAF1 and ASNA1. KM-DD as well as the truncation lacking the MSP domain (C-Ter) are defective in binding poly-ubiquitinated proteins and seem to interact preferentially with oligo-ubiquitinated proteins. **(F)** The binding of ubiquitinated proteins to VAPB is reduced in cells treated with ASNA1 siRNA. U2OS cells expressing Flag-VAPB from a tetracycline-inducible promoter were treated with the indicated siRNA oligos. Depletion of ASNA1 reduces ubiquitin and BAG6 binding, but not FAF1 binding, to Flag-VAPB. C-Ter, C-terminal half; IP, immunoprecipitate; KM-DD, K87D M89D double mutant; Luc, luciferase.

As indicated above, VAPB did not appear to be targeted for proteasomal degradation. However, upon Flag-VAPB immunoprecipitation from human cells expressing VAPB from a tetracycline-inducible promoter, we found that VAPB interacted with ubiquitinated proteins and the interaction was stimulated upon proteasome inhibition with MG132 (Figure 4A). Interestingly, proteasome inhibition also stimulated VAPB interaction with FAF1 and p97 (Figure 4A). Similar results were obtained when we immunoprecipitated endogenous VAPB using specific antibodies (Figure 4B). These data suggested that although VAPB is not a proteasome target itself, it can interact with proteins that are ubiquitinated and destined for proteasome-mediated degradation.Because ubiquitin and FAF1 binding to VAPB appeared to correlate, and because FAF1 is a ubiquitin-binding protein, we next tested whether FAF1 was required for VAPB interaction with ubiquitinated proteins. We found that the binding of ubiquitinated proteins was strongly reduced in Flag-VAPB immunoprecipitates from cells treated with four independent siRNA oligos for FAF1 compared to cells treated with no siRNA or luciferase siRNA (Figure 4C). Similarly, the binding of ubiquitinated proteins to endogenous VAPB was reduced upon FAF1 depletion (Figure 4D). These data suggest that FAF1 might facilitate, at least in part, the binding of ubiquitinated proteins to VAPB. Furthermore, the VAPB double mutant K87D M89D (KM-DD) as well as the truncated form of VAPB lacking the MSP domain (C-Ter) are not only defective in FFAT and FAF1 binding, but also in ubiquitin binding (Figure 4E). These VAPB variants seem to preferentially bind proteins carrying shorter ubiquitin chains than wild-type VAPB, raising the interesting possibility that FAF1 might, in fact, be required to protect the ubiquitin chains from deubiquitinating enzymes or from premature degradation. For comparison, we include a long exposure of a Flag Western blot to demonstrate that the different pattern observed in the ubiquitin blot is not due to differences in the migration of Flag-VAPB itself. The prominent band that can be detected half way between 50 and 75 kDa upon long exposure of anti-Flag blots, we presume to represent VAPB dimers that resist SDS denaturation.

### Mass spectrometry analysis of VAPB immunoprecipitates reveals VAPB interaction with the TRC subunits

In an attempt to identify the ubiquitinated proteins that interact with VAPB, we used the stable isotope labeling by amino acids in cell culture (SILAC) technique of mass spectrometry on endogenous VAPB immunoprecipitates to search for proteins that accumulate after 2 and 6 hr of proteasome inhibition with MG132. As expected, several OSBPs and other FFAT proteins were identified in VAPB immunoprecipitates, but their light/heavy SILAC ratios were often close to 1 and did not follow a pattern consistent with accumulation in the MG132 treated samples, i.e. ratios higher than 1 when the light samples were treated with MG132 and ratios lower than 1 when the heavy samples were treated with MG132 (Additional file 3: Table S2 and Additional file 4: Table S3). Hence, their binding to VAPB appeared to be unaffected by proteasome inhibition. In general, we noticed that proteasome inhibition caused only a mild accumulation of some VAPB-interacting partners, with ratios mostly below twofold. Among these, our attention was drawn to the four subunits of the TRC – ASNA1, BAG6, UBL4A and GET4 – that were all present in VAPB immunoprecipitates and followed a similar trend of slightly accumulating upon proteasome inhibition (Table 2, Additional file 4: Table S3). Our interest was further strengthened by the observation that all four TRC subunits were also identified by mass spectrometry in Flag-FAF1 immunoprecipitates (Table 3, Additional file 5: Table S4). One subunit in particular, ASNA1 (also known as TRC40 or GET3), appeared to be more abundant in Flag-FAF1 immunoprecipitates from cells treated with MG132. Hence, we went on to evaluate ASNA1 as a potential ubiquitinated target that VAPB and FAF1 might have in common.

**Table 2:** TRC complex subunits slightly accumulate in endogenous VAPB immunoprecipitates upon proteasome inhibition

**Protein name**	**UniProt ID**	**MW (Da)**	**SILAC ratio L/H**^**a**^			
			**L + MG 2 hr**	**H + MG 2 hr**	**L + MG 6 hr**	**H + MG 6 hr**
ASNA1	O43681	38,793	1.31 ± 0.08 (31)	0.79 ± 0.07 (27)	1.42 ± 0.10 (32)	0.87 ± 0.06 (35)
BAG6	P46379	119,409	1.47 ± 0.18 (36)	0.77 ± 0.11 (36)	1.43 ± 0.19 (52)	0.94 ± 0.10 (43)
GET4	Q7L5D6	36,504	1.39 ± 0.11 (8)	0.73 ± 0.06 (11)	1.30 ± 0.08 (9)	0.84 ± 0.03 (13)
UBL4A	P11441	17,777	1.50 ± 0.10 (7)	0.74 ± 0.04 (9)	1.51 ± 0.15 (7)	0.89 ± 0.04 (10)

^a^Light- (L) and heavy-labeled (H) cells were treated with MG132 (MG) for 2 or 6 hr, as indicated. Equal amounts of light- and heavy-labeled extracts were mixed and endogenous VAPB was immunoprecipitated using specific antibodies. The light/heavy SILAC ratios (L/H) determined by mass spectrometry are indicated. L + MG indicates that the light-labeled samples were treated with MG132. Proteins whose interaction with VAPB is stimulated by proteasome inhibition accumulate in these samples, resulting in L/H ratios higher than 1. H + MG indicates that the heavy-labeled samples were treated with MG132. Protein accumulation in the heavy-labeled samples results in L/H ratios lower than 1. The L/H for proteins that are not affected by proteasome inhibition will be close to 1. The numbers in parenthesis represent the number of spectra analyzed for each sample.

**Table 3:** TRC subunits were identified by mass spectrometry in Flag-FAF1 immunoprecipitates

**Protein name**	**UniProt ID**	**MW (Da)**	**Share of spectrum IDs**^**a**^		
			**No MG**	**MG 2 hr**	**MG 6 hr**
ASNA1	O43681	38,793	0.11%	0.30%	0.18%
BAG6	P46379	119,409	0.50%	0.56%	0.49%
GET4	Q7L5D6	36,504	0.05%	0.10%	0.08%
UBL4A	P11441	17,777	0.11%	0.13%	0.08%

^a^Flag-FAF1 was immunoprecipitated from U2OS cells treated with MG132 (MG) for 0, 2 or 6 hr as indicated. The share of spectrum IDs is indicated as a measure of protein abundance in the immunoprecipitates.

### ASNA1 is a novel FFAT-like binding partner of VAPB

**Figure 5:**
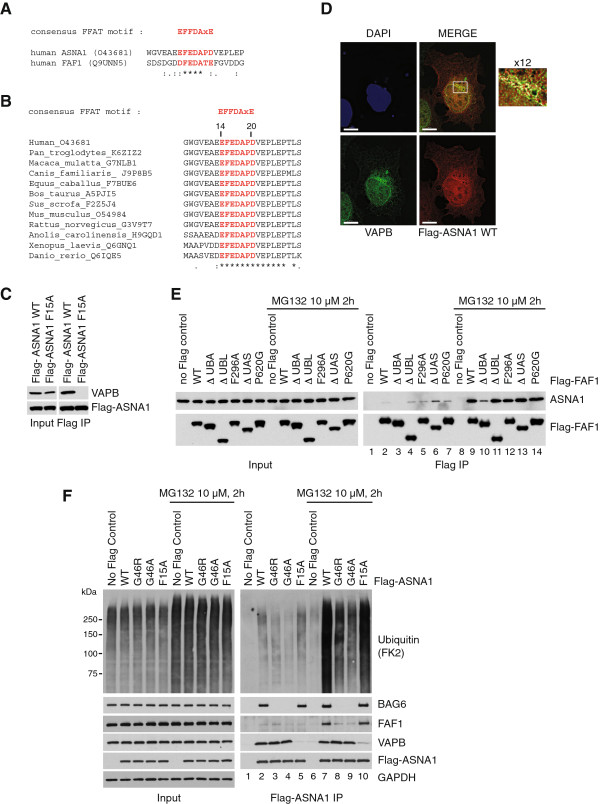
**ASNA1 interacts with VAPB via a FFAT-like motif similar to FAF1. (A)** Alignment of the FFAT-like motifs in human ASNA1 and FAF1. **(B)** Alignment of the FFAT-like motif of ASNA1 across species showing that it is highly conserved. **(C)** A point mutation in the FFAT-like motif of ASNA1 (F15A) abolishes its interaction with VAPB. WT and mutant Flag-ASNA1 were immunoprecipitated from U2OS cells using anti-Flag beads. **(D)** Indirect immunofluorescence of VAPB and Flag-ASNA1 WT. U2OS cells were transfected with Flag-ASNA1 WT for 24 hr. Flag-ASNA1 WT (red) is co-localized with VAPB (green) in a peri-nuclear area (enlarged window) suggesting an ER pattern. Scale bar is 10 μm. **(E)** ASNA1 interaction with FAF1 is strongly stimulated upon proteasome inhibition with MG132 and depends on the UBA domain. WT Flag-FAF1 and the indicated mutants were immunoprecipitated from U2OS cells. **(F)** G46R and G46A point mutations in ASNA1 abolish its interaction with BAG6 and strongly reduce its interaction with FAF1 and ubiquitin, most noticeably after MG132 treatment, but do not affect the interaction with VAPB. WT and mutant Flag-ASNA1 were immunoprecipitated from SH-SY5Y cells. DAPI, 4',6-diamidino-2-phenylindole; IP, immunoprecipitate; WT, wild type.

To begin with, we confirmed by Western blotting that ASNA1 is present in Flag-VAPB (Figure 4A) and endogenous VAPB immunoprecipitates from human cells (Figure 4B). Consistent with the SILAC mass spectrometry results, we observed a very mild increase in ASNA1 binding to endogenous VAPB upon MG132 treatment (Figure 4B). No such change could be observed in Flag-VAPB immunoprecipitates (Figure 4A). Moreover, ASNA1 levels did not change upon proteasome inhibition (Figure 4A,B, left panels). Taken together, these data suggested that ASNA1 may not be itself a proteasome target. However, because ASNA1 interaction with VAPB was fairly robust, we proceeded to search for a potential FFAT motif in any of the TRC subunits that could mediate a direct interaction with VAPB. This led to the identification of a FFAT-like sequence close to the N-terminus of ASNA1 that was very similar to the one we uncovered in FAF1 (Figure 5A). As seen before for FAF1, this region of ASNA1 is well conserved across species (Figure 5B). Indeed, mutating the phenylalanine residue (Phe15 to Ala) in this putative FFAT motif abolished Flag-ASNA1 interaction with VAPB (Figure 5C). Reciprocally, the VAPB double mutant (K87D M89D) that is defective in FFAT binding was unable to interact with ASNA1 (Figure 4E). Like FAF1, ASNA1 interacts efficiently with the MSP domain, but not with the C-terminal region of VAPB (Figure 4E). We conclude that ASNA1 interacts with the MSP domain of VAPB via a FFAT-like motif similar to FAF1. As seen before for FAF1, Flag-ASNA1 co-localization with endogenous VAPB is most prominent in the peri-nuclear region (Figure 5D).

Because the TRC complex has been implicated in ubiquitin-dependent degradation of mislocalized ER membrane proteins [35], we also checked if ASNA1 might contribute to the binding of ubiquitinated proteins to VAPB. As shown in Figure 4F, ASNA1 depletion by siRNA leads to a reduction in Flag-VAPB interaction with ubiquitinated proteins. We conclude that part of the ubiquitinated targets that interact with VAPB could be TRC quality control targets that are recruited via ASNA1.

### FAF1 interaction with ASNA1 depends on the UBA domain

Next we wanted to verify whether ASNA1 also interacts with FAF1 as suggested by the mass spectrometry analysis (Table 3). Unlike ASNA1 interaction with VAPB, its interaction with Flag-FAF1 was weak and hard to detect in the absence of proteasome inhibition (Figure 5E, lanes 2 to 7). Upon proteasome inhibition, ASNA1 interaction with Flag-FAF1 became readily detectable (Figure 5E, lanes 9 to 14) and it required the UBA domain of FAF1 (Figure 5E, lane 10). Conversely, Flag-ASNA1 interaction with endogenous FAF1 was only detectable upon proteasome inhibition, which also caused a considerable increase in the binding of ubiquitinated proteins to ASNA1 (Figure 5F, compare lanes 2 and 7). Interestingly, ASNA1 interaction with FAF1 did not depend on the ASNA1 FFAT-motif (Figure 5F, lane 10), but was drastically reduced by a Gly46 point mutation in ASNA1 to either Ala or Arg (Figure 5F, lanes 8 and 9) [36]. We found that these two mutants are completely defective in interacting with the BAG6 subunit of the TRC complex and exhibit reduced binding to ubiquitinated proteins (Figure 5F). Taken together, these findings indicate a correlation between the binding of ubiquitinated proteins to ASNA1 and a detectable interaction with FAF1. They suggest that FAF1 binding to ASNA1 is likely indirect and mediated by the ubiquitinated proteins that associate with ASNA1.

### RPN2 is a common ubiquitinated target for VAPB and FAF1

**Figure 6:**
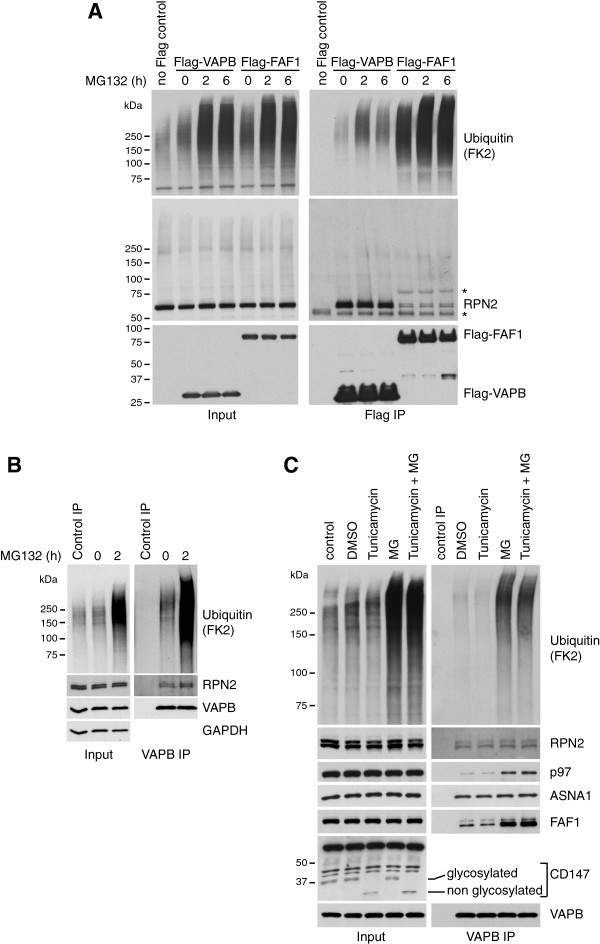
**RPN2 is a common interactor of VAPB and FAF1. (A)** Flag-VAPB and Flag-FAF1 were immunoprecipitated from U2OS cells treated with 10 μM MG132 for 2 hr, 5 μM MG132 for 6 hr or left untreated (0 hr). Asterisks indicate non-specific bands. **(B)** Proteasome inhibition enhances RPN2 binding to endogenous VAPB. Endogenous VAPB was immunoprecipitated from U2OS cells treated with 10 μM MG132 for 2 hr or left untreated (0 hr). **(C)** ER stress induced by tunicamycin treatment does not affect RPN2 levels or its binding to VAPB. Endogenous VAPB was immunoprecipitated from U2OS cells treated with 5 μg/ml tunicamycin for 5 h, 10 μM MG132 for 2 hr, both tunicamycin and MG132, or dimethyl sulfoxide as a control. DMSO, dimethyl sulfoxide; IP, immunoprecipitate; MG, MG132.

Because our SILAC mass spectrometry analysis of endogenous VAPB immunoprecipitates did not reveal any protein that would significantly accumulate upon proteasome inhibition, we reasoned that the ubiquitinated targets of VAPB might be present at very low levels in the immunoprecipitates and escape our analysis. To overcome this limitation, we decided to enrich the ubiquitinated peptides prior to mass spectrometry analysis using a two-step immunoprecipitation protocol. A mixture of light-labeled Flag-VAPB and heavy-labeled Flag-FAF1 immunoprecipitates was trypsin-digested and the peptides carrying a di-glycine signature were isolated using antibodies specific to lysine-ϵ-GlyGly [37]. Di-glycine peptides mark the site of ubiquitination in the protein of origin. They have low abundance relative to peptides that do not carry a GlyGly extension and they are also harder to detect by mass spectrometry. Di-glycine peptide enrichment using specific antibodies greatly enhances their identification by mass spectrometry and unequivocally demonstrates which proteins identified in our immunoprecipitates were modified with ubiquitin. To specifically identify ubiquitinated targets common for VAPB and FAF1, the list of ubiquitinated proteins obtained from the mass spectrometry analysis of di-glycine peptides was cross-referenced with proteins we previously identified by mass spectrometry in both Flag-VAPB and Flag-FAF1 immunoprecipitates (Additional file 6: Table S5). This revealed two potentially interesting targets, the RPN2 and DDOST subunits of the N-oligosaccharyl-transferase (Table 4), which, like VAPB, are localized at the ER. Both proteins appeared to accumulate slightly in endogenous VAPB immunoprecipitates upon proteasome inhibition, as determined by SILAC mass spectrometry (Table 5, Additional file 4: Table S3) and were identified at low levels in Flag-FAF1 immunoprecipitates (Table 6, Additional file 5: Table S4). We confirmed RPN2 interaction with Flag-VAPB and Flag-FAF1 by Western blotting and found the interaction with VAPB to be more robust (Figure 6A). There was no detectable change in RPN2 levels upon proteasome inhibition (Figure 6A, left panel), nor could we detect ubiquitinated forms of RPN2 in extracts or immunoprecipitates (Figure 6A). This suggests that only a minor fraction of RPN2 is subject to ubiquitin-mediated proteasomal degradation. However, we could detect a modest increase in RPN2 binding to endogenous VAPB (Figure 6B, right panel), consistent with the mass spectrometry results (Table 5). This supports the notion that VAPB interacts with a fraction of RPN2 that is destined for proteasome-mediated degradation. If RPN2 were a target for the ERAD quality control pathway, we reasoned that artificially inducing ER stress with tunicamycin might stimulate RPN2 degradation and its association with VAPB and FAF1. We found that tunicamycin treatment had no significant effect on RPN2 levels or its interaction with endogenous VAPB (Figure 6C). Furthermore, tunicamycin did not stimulate VAPB interaction with FAF1 and there was no added effect of tunicamycin when used in combination with MG132. In contrast, another glycosylated protein, CD147, shifted from the glycosylated form to a faster migrating, non-glycosylated form, indicating that the tunicamycin treatment was effective at least to some extent (Figure 6C, left panel). We are not certain whether our ER-stress conditions are not optimal to affect RPN2 or RPN2 ubiquitin-dependent degradation is part of its regulation and not a quality control process.

**Table 4:** Ubiquitinated targets of VAPB and FAF1 identified by mass spectrometry upon enrichment of ubiquitinated peptides

**Protein name**	**UniProt ID**	**GlyGly peptides**^**a **^**(times identified)**^**b**^
RPN2	P04844	LS**K**^**154**^EETVLATVQALQTASHLSQQADLR (4×)
		**K**^**244**^NFESLSEAFSVASAAAVLSHNR (2×)
		LHNQ**K**^**442**^TGQEVVFVAEPDNK (3×)
DDOST	P39656	APTIVG**K**^**189**^SSLNPILFR (2×)

^a^A mixture of light-labeled Flag-VAPB and heavy-labeled Flag-FAF1 immunoprecipitates was analyzed by mass spectrometry after ubiquitinated peptide enrichment using antibodies specific to lysine-ϵ-GlyGly. The lysine residues carrying the GlyGly modification are indicated in bold and the superscript is the position in the protein sequence.

^b^The numbers in parenthesis indicate how many times each peptide was identified by mass spectrometry.

**Table 5:** RPN2 and DDOST slightly accumulate in endogenous VAPB immunoprecipitates upon proteasome inhibition

**Protein name**	**UniProt ID**	**MW (Da)**	**SILAC ratio L/H**^**a**^			
			**L + MG 2 hr**	**H + MG 2 hr**	**L + MG 6 hr**	**H + MG 6 hr**
RPN2	P04844	69,284	1.11 ± 0.02 (4)	0.88 ± 0.00 (2)	1.30 ± 0.10 (4)	0.96 ± 0.02 (6)
DDOST	P39656	50,801	-	-	1.12 ± 0.00 (2)	0.64 ± 0.00 (1)

^a^Light- (L) or heavy-labeled (H) cells were treated with MG132 (MG) for 2 or 6 hr, as indicated. Equal amounts of light- and heavy-labeled extracts were mixed, and endogenous VAPB was immunoprecipitated using specific antibodies. The light/heavy SILAC ratios (L/H) determined by mass spectrometry are indicated. L + MG indicates that the light-labeled samples were treated with MG132. Proteins whose interaction with VAPB is stimulated by proteasome inhibition accumulate in these samples, resulting in L/H ratios higher than 1. H + MG indicates that the heavy-labeled samples were treated with MG132. Protein accumulation in the heavy-labeled samples results in L/H ratios lower than 1. The L/H for proteins that are not affected by proteasome inhibition will be close to 1. The numbers in parenthesis represent the number of spectra analyzed for each sample.

**Table 6:** RPN2 and DDOST were identified at low levels in Flag-FAF1 immunoprecipitates

**Protein name**	**UniProt ID**	**MW (Da)**	**Share of spectrum IDs**^**a**^		
			**No MG**	**MG 2 hr**	**MG 6 hr**
RPN2	P04844	69,284	-	0.05%	0.03%
DDOST	P39656	50,801	0.04%	0.05%	0.04%

^a^Flag-FAF1 was immunoprecipitated from U2OS cells treated with MG132 (MG) for 0, 2 or 6 hr, as indicated. The share of spectrum IDs is indicated as a measure of protein abundance in the immunoprecipitates.

### VAPA and VAPB interact with proposed FFAT proteins, RAB3GAP1 and WDR44

**Figure 7:**
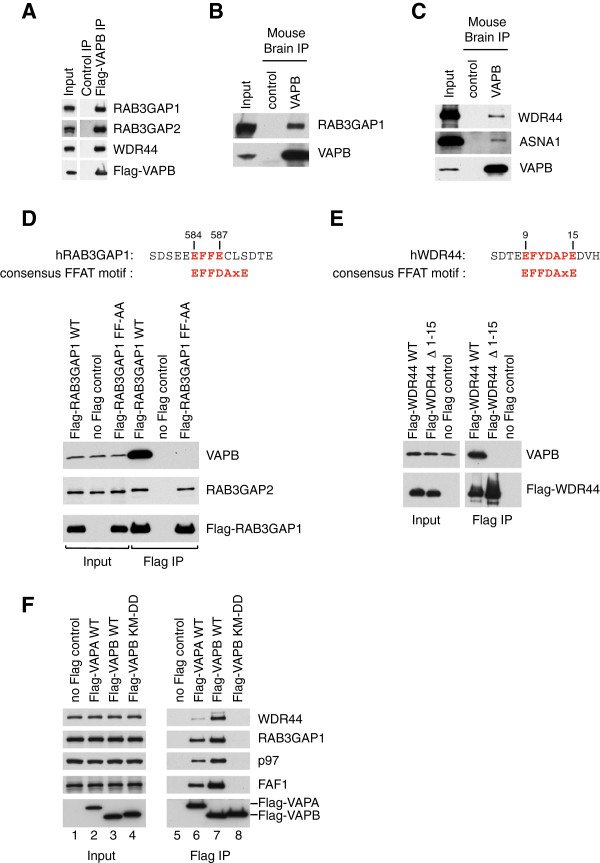
**RAB3GAP1 and WDR44 are FFAT-like interactors of VAPB. (A)** Flag-VAPB immunoprecipitated from HeLa cells interacts with RAB3GAP1, RAB3GAP2 and WDR44. The RAB3GAP1 and WDR44 interactions with VAPB are mediated by FFAT motifs. VAPB immunoprecipitation from mouse brain extract confirms its interaction with RAB3GAP1 **(B)**, ASNA1 and WDR44 **(C)**. Flag-RAB3GAP1 WT and F585A/F586A (FF-AA) **(D)** and Flag-WDR44 WT and Δ1-15 **(E)** were immunoprecipitated from U2OS cells. Both RAB3GAP1 and WDR44 contain FFAT motifs, as indicated in the top panels, which are responsible for the interaction with VAPB. The formation of the RAB3GAP1/2 heterodimer is not affected by the mutation of the FFAT motif in RAB3GAP1. **(F)** Immunoprecipitation of Flag-VAPA WT, Flag-VAPB WT or the K87D M89D mutant of VAPB from U2OS cells shows that the KM-DD mutant is defective in interacting with RAB3GAP1 and WDR44. FF-AA, F585A/F586A; IP, immunoprecipitate.

Our mass spectrometry analysis of Flag-VAPA, Flag-VAPB and endogenous VAPB immunoprecipitates also revealed three binding partners for VAPA and VAPB that appeared to be very abundant, especially in Flag immunoprecipitates. These were RAB3GAP1, RAB3GAP2 and WDR44 (Table 7, Additional file 7: Table S6, Additional file 8: Table S7 and Additional file 9: Table S8). RAB3GAP1 and 2 form a GTPase activating complex for RAB3 proteins [38,39], which are expressed specifically in the brain and regulate neurotransmitter release [40]. First, we set out to confirm these interactions by Western blotting. Indeed, Flag-VAPB expressed in HeLa cells co-immunoprecipitated RAB3GAP1 and 2, as well as WDR44 (Figure 7A). Furthermore, endogenous VAPB from mouse brain co-immunoprecipitated RAB3GAP1 (Figure 7B) as well as WDR44 and ASNA1 (Figure 7C), suggesting that these interactions could be relevant for VAPB function in the brain. The seemingly strong interaction these proteins exhibited with VAPA and VAPB prompted us to have a closer look at their primary sequence to check if they might harbor a FFAT motif. There was no such motif in RAB3GAP2, but RAB3GAP1 did contain a short sequence that partly resembled a FFAT motif (Figure 7D). Mutating both phenylalanine residues to alanine fully prevented Flag-RAB3GAP1 interaction with VAPB and had no effect on its interaction with RAB3GAP2 (Figure 7D). We conclude that, although non-canonical, the short motif present in RAB3GAP1 is a bona fide FFAT motif in that it mediates the interaction with VAPA/B. WDR44, on the other hand, contained a putative FFAT motif that closely resembled those found in OSBPs (Additional file 3: Table S2) and was located close to the N-terminus of the protein. A truncated version of WDR44 lacking the first 15 amino acids was unable to interact with VAPB (Figure 7E), confirming that this was the region that mediated VAPB binding. Furthermore, we showed that the VAPB double mutant defective in FFAT binding (K87D M89D) was not only defective in FAF1 binding, but was also unable to interact with RAB3GAP1 or WDR44 (Figure 7F, compare lanes 7 and 8). Hence, we conclude that FAF1, RAB3GAP1 and WDR44, all interact in a similar manner with the MSP domain of VAPA and VAPB.

**Table 7:** FFAT-like proteins identified in VAPA and VAPB immunoprecipitates by mass spectrometry

**Protein name**	**UniProt ID**^**a**^	**MW (Da)**^**a**^	**FFAT motif**^**a**^	**Share of spectrum IDs**^**b**^			
				**Flag-VAPA (U2OS)**	**Flag-VAPB (U2OS)**	**Endogenous VAPB**	
						**HeLa cells**	**Mouse brain**
FAF1	Q9UNN5	73954	DFEDATE	0.85%	0.68%	0.06%	-
ASNA1	O43681	38793	EFEDAPD	0.69%	0.79%	0.20%	-
RAB3GAP1	Q15042	110524	EFFECLS	2.65%	1.79%	0.20%	0.43%
RAB3GAP2	Q9H2M9	155985	NA	1.73%	1.61%	0.10%	0.37%
WDR44	Q5JSH3	101366	EFYDAPE	1.97%	1.66%	-	0.05%
*STX1B*^c^	*P61266*	*33245*	*EFFEQVE*	*-*	*-*	*-*	*0.20*%
*STX1A*^c^	*Q16623*	*33023*	*EFFEQVE*	*-*	*-*	*-*	*0.03*%

^a^The UniProt ID, MW and FFAT motif are indicated for the human proteins.

^b^The share of spectrum IDs is indicated as a measure of protein abundance in the immunoprecipitates.

^c^STX1A and B are shown in italics because their putative FFAT-like motifs could not be confirmed experimentally.

NA, not applicable.

Our analysis of VAPB immunoprecipitates from mouse brain also revealed VAPB interaction with syntaxin 1A and B (STX1A and B) (Table 7, Additional file 9: Table S8, Additional file 10: Figure S2A). These proteins harbor a sequence that resembles a FFAT motif (Additional file 10: Figure S2B), but mutating the two phenylalanine residues in either STX1A or STX1B to alanine had no effect on their interaction with VAPB (Additional file 10: Figure S2C). Hence, these are not functional FFAT motifs and are not responsible for syntaxin 1A/B interaction with VAPB.

## Discussion

### FAF1 is a FFAT-like binding partner for VAPB

Humans express five ubiquitin-binding UBX proteins, including FAF1 and UBXD7. Previous work showed that UBXD7 is unique among p97 cofactors due to the presence of an UIM motif that allows it to interact directly with neddylated cullins [10,11]. Here we show that FAF1 contains an atypical FFAT motif that allows it to associate with the membrane-anchored proteins VAPA and VAPB. Although distinct from the consensus proposed for FFAT motifs [27] due to the absence of a second aromatic residue, the FFAT motif of FAF1 is still able to mediate the interaction with the MSP domain of VAPB.

The UIM motif of UBXD7 and the FFAT motif of FAF1 represent features unique to each of these UBX proteins and mediate their interaction with specific targets, neddylated cullins and VAPA/B respectively. The identification of these short protein-interaction motifs supports the notion that the general function of UBA-UBX proteins as ubiquitin-binding adaptors for p97 is complemented with specific functions mediated by singular motifs.

### Identification of novel binding partners of VAPB carrying FFAT-like sequences

The identification of a degenerated, yet functional FFAT motif in FAF1, indicated that other proteins carrying FFAT-like motifs could represent bona fide interaction partners for VAPB. On one hand, non-canonical FFAT motifs may be present in known VAPB-binding partners and on the other hand, searching for FFAT motifs that only partially match the consensus sequence could lead to the identification of novel VAPB-binding partners.

We took the first approach and validated the FFAT-like motifs in ASNA1, RAB3GAP1 and WDR44, proteins that we identified in VAPB immunoprecipitates by mass spectrometry. Mikitova and Levine took the latter approach and computationally identified multiple high-confidence FFAT-like proteins, based on the degree of variation that is tolerated by FFAT motifs in a yeast reporter assay [41]. RAB3GAP1 scored high as a potential binding partner for VAPB in this study, but the more divergent FFAT-like motifs in FAF1 and ASNA1 were not recognized.

FFAT-like motifs of WDR44 and RAB3GAP1 have been picked out by prior computational searches [27,28,41] and we confirm that they are functional and truly mediate VAPB binding. Our experiments with syntaxin 1A and B, whose FFAT-like sequences are not required for the interaction with VAPB, further highlight the importance of experimentally testing putative FFAT motifs.

### VAPB interacts with ubiquitinated proteins

Several observations led us to believe that VAPB itself is not a proteasome substrate: (1) VAPB does not accumulate upon proteasome inhibition with MG132 (Figure 4B); (2) anti-Flag Western blots of Flag-VAPB immunoprecipitates did not reveal any slower migrating, ubiquitinated forms of VAPB, even after proteasome inhibition (Additional file 2: Figure S1); (3) VAPB interaction with FAF1 is not mediated by the ubiquitin-binding UBA domain of FAF1 (Figure 2B), and (4) no ubiquitinated peptide was identified for VAPB in our mass spectrometry analysis of di-glycine peptides from Flag-VAPB/Flag-FAF1 immunoprecipitates.

Kanekura *et al.* previously observed ubiquitinated species in over-expressed VAPB immunoprecipitates and assumed them to be ubiquitinated VAPB [34]. Instead, we present evidence for VAPB interaction with ubiquitinated proteins: (1) VAPB interacts with ubiquitinated proteins as shown by the anti-ubiquitin Western blotting of Flag-VAPB and endogenous VAPB immunoprecipitates; (2) VAPB interacts directly with a ubiquitin-binding protein, FAF1, and (3) VAPB interaction with ubiquitin is reduced when FAF1 is depleted by treatment with siRNA (Figure 4).One possibility is that the direct binding of FAF1 to VAPB mediates VAPB interaction with ubiquitinated species that are recognized by the UBA domain of FAF1. Alternatively, the proteins that are modified with ubiquitin interact directly with VAPB and FAF1, which is required to protect the ubiquitin chains from deubiquitinating enzymes or from degradation via alternative ubiquitin receptors. This latter possibility is strongly supported by the observation that VAPB variants that cannot bind FFAT proteins interact with ubiquitinated species carrying shorter ubiquitin chains than observed for wild-type VAPB (Figure 4E). Furthermore, RPN2, a novel ubiquitinated target we identified for VAPB and FAF1, interacts more robustly with VAPB than with FAF1 (Figure 6A), suggesting that the interaction is unlikely to be mediated by FAF1.

### FFAT-like proteins add to the complexity of VAPB function

**Figure 8:**
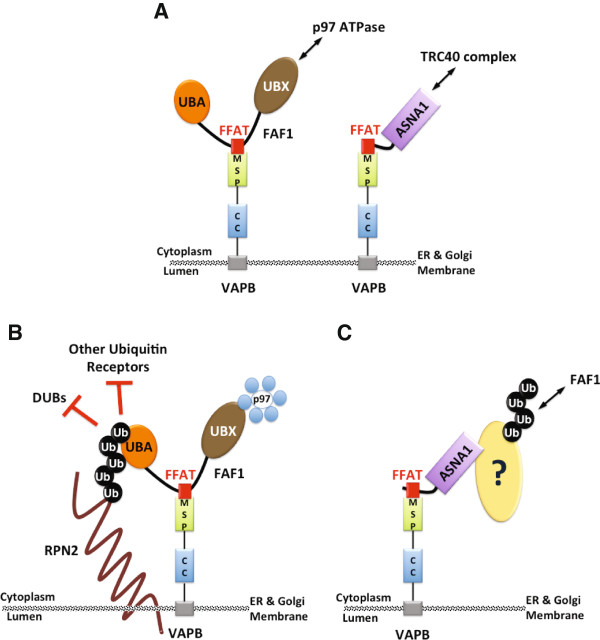
**VAPB interacts with novel FFAT-like proteins, FAF1 and ASNA1. (A)** The FFAT-like motif of FAF1 mediates its interaction with the MSP domain of VAPB and recruits FAF1/p97 to the ER membrane. Similarly, the FFAT-like sequence in ASNA1 mediates VAPB interaction with the TRC complex. **(B)** RPN2 is a common ubiquitinated target for FAF1 and VAPB. We propose that, on one side, FAF1 protects ubiquitinated RPN2 from deubiquitinating enzymes (DUBs) and other ubiquitin receptors and, on the other side, it recruits p97 hexamers that are necessary for extracting misfolded or misassembled RPN2 from the ER membrane, to allow for its proteasome-mediated degradation. **(C)** ASNA1 mediates VAPB interaction with a subset of ubiquitinated targets that interact with FAF1 via their ubiquitin chains. We propose that VAPB might represent an alternative receptor for TRCs that are implicated in clearing mislocalized ER proteins.

Most, if not all, canonical FFAT proteins identified to date are involved in lipid metabolism. They include lipid-binding proteins such as the OSBPs and lipid-transport proteins such as the PITPNMs and the ceramide transfer protein (CERT) [26]. Here we show that proteins with slightly divergent FFAT motifs can also interact with the MSP domain of VAPB. These novel interaction partners add further layers of complexity to the VAPB function by implicating VAPB in p97-regulated processes via FAF1, in TRC complex function via ASNA1 and in RAB3 regulation via RAB3GAPs.The related FFAT motifs present in FAF1 and ASNA1 link VAPB to ATPase complexes, such as p97 and the TRC (Figure 8A). The identification of RPN2 and DDOST as common ubiquitinated targets for VAPB and FAF1 suggests that VAPB might recruit FAF1/p97 to ubiquitinated targets located at the ER membrane (Figure 8B). Both RPN2 and DDOST are mostly lumenal and anchored in the ER membrane via C-terminal transmembrane regions. Interestingly, all the ubiquitination sites we identified in RPN2 and DDOST are located in the lumenal region of the proteins, highlighting the importance of the interaction with the p97 machinery for retrotranslocation from the ER into the cytosol, where they would become accessible to ubiquitin ligases and the proteasome.

ASNA1, on the other hand, mediates VAPB interaction with TRC, which is required for membrane insertion of some tail-anchored proteins [36] and also for the elimination of mislocalized membrane proteins [35]. Interestingly, although VAPB is a tail-anchored protein, it does not interact with the TRC via its C-terminal transmembrane region. We show that ASNA1 interacts only with the MSP domain of VAPB and not with the C-terminal region, which includes the transmembrane domain (Figure 4E). CAML and WRB were identified to be the receptors that recruit the TRC to the ER membrane for insertion of tail-anchored proteins [42,43]. We show that ASNA1 transiently interacts with ubiquitinated proteins that are targeted for proteasomal degradation and appear to recruit FAF1 (Figure 5F). Furthermore, a fraction of the ubiquitinated proteins that interact with VAPB are recruited via ASNA1 (Figure 4F). It is possible that VAPB may represent an alternative receptor for TRCs whose ubiquitinated targets require p97/FAF1 for degradation (Figure 8C).

## Conclusions

Starting from the observation that the p97 cofactor FAF1 interacts with the ER membrane proteins VAPA and VAPB, we identified an atypical FFAT sequence in FAF1 that allows its direct interaction with the MSP domain of VAPA/B. A similar FFAT-like sequence was subsequently recognized in the ASNA1 subunit of the TRC, further expanding the repertoire of proteins that interact with VAPB in this manner. The identification of two subunits of an ER-resident glycosyltransferase – RPN2 and DDOST – as common ubiquitinated targets for VAPB and FAF1, suggests that VAPB might recruit p97/FAF1 to facilitate degradation of a subset of targets located at the ER.

Thus, VAPB dimers emerge as a platform for docking various FFAT and FFAT-like proteins at the ER membrane. These include proteins involved in lipid metabolism as previously noted, but also, as shown in this study, the ubiquitin receptor FAF1, the ASNA1 component of the TRC that mediates/monitors protein insertion in the ER membrane and regulatory enzymes such as RAB3GAP1/2. Perturbations in VAPB interaction with any of these proteins when VAPB aggregates due to the ALS-causing P56S mutation could be responsible for the defects that lead to ALS8 disease.

## Methods

### Cloning information

Human FAF1 [GenBank:NM_007051.2] was amplified from EST IMAGE 5928559. For mammalian expression, wild-type and mutant FAF1 variants (lacking the native methionine) were subcloned as Sal1/Not1 inserts into pCMV5-Flag. For bacterial expression, wild-type FAF1 was subcloned into a modified pGEX6P-1 vector containing a TEV protease site and a Flag-tag downstream of the GST. Human VAPB [GenBank:NM_004738.3] was amplified from EST IMAGE 3543354. For mammalian expression, wild-type, mutant and truncated variants were subcloned as BamH1/Not1 inserts into either pCMV5-Flag or a modified version of pcDNA5-FRT/TO containing an N-terminal Flag-tag. For bacterial expression, ORFs were subcloned as BamH1/Not1 inserts into a modified pGEX6P-1 vector containing a TEV protease site, before removing extraneous linker residues between the glutamine of the TEV and the native methionine of VAPB by site-directed mutagenesis. Human VAPA [GenBank:NM_003574] was amplified from EST IMAGE 4662097 and subcloned as a BamH1/Not1 insert into pCMV5-Flag for mammalian expression. Human RAB3GAP1 [GenBank:NM_001172435.1] was amplified from EST IMAGE 5276867. For mammalian expression, a silent mutation (t909c) was first introduced to disrupt an internal BamH1 site before subcloning as BamH1/Not1 inserts into pcDNA5-FRT/TO-Flag. Human WDR44 [GenBank:NM_019045.4] was amplified by RT-PCR from embryonic umbilical RNA (Agilent Technologies, Wokingham, UK). Human ASNA1 [GenBank:NM_004317.2] was amplified by RT-PCR from total uterus RNA (Agilent Technologies). Wild-type and mutant variants were subcloned as BamH1/Not1 inserts into pCMV5-Flag for mammalian expression.

PCR reactions were carried out using KOD Hot Start DNA Polymerase (Merck Millipore, Darmstadt, Germany). Reverse transcriptase reactions were carried out using Superscript III (Life Technologies). All full-length products were cloned into pSc-B or pSc-A (Agilent Technologies, Carlsbad, USA) and fully sequenced prior to further subcloning or manipulation. All mutations and deletions were made following the Quickchange method (Agilent Technologies), but using KOD Hot Start DNA Polymerase. DNA sequencing was performed by the Sequencing Service at the College of Life Sciences, University of Dundee [44]. For plasmid requests, please contact Medical Research Council Protein Phosphorylation and Ubiquitylation Unit (MRC-PPU) Reagents [45].

### Cell culture and transfection

U2OS and HeLa cells were respectively cultured in McCoy’s and MEM media (Gibco, Pailsey, UK) supplemented with 10% fetal bovine serum (FBS) (Thermo Scientific, Fremont, USA), 2 mM L-glutamine (Invitrogen, Camarillo, USA), 100 units/ml penicillin, 100 μg/ml streptomycin (Invitrogen). SH-SY5Y cells were cultured in DMEM/F12 (Gibco) supplemented with 10% FBS, 1% NEAA (Gibco), 2 mM L-glutamine, 100 units/ml penicillin, 100 μg/ml streptomycin. Then 10 μM MG132 (Enzo, Farmingdale, USA) was added to the media for the indicated time before harvesting the cells. Tunicamycin (Sigma, Saint Louis, USA) was used at 5 μg/ml for 5 hr.

Flag-VAPB and Flag-FAF1 U2OS cells were generated from Flp-In T-REx U2OS cells, following Invitrogen’s instructions and were maintained in McCoy’s media supplemented with 10% FBS, 2 mM L-glutamine, 100 units/ml penicillin, 100 μg/ml streptomycin, 100 μg/ml hygromycin, 15 μg/ml blasticidin (Invitrogen). The expression of Flag-VAPB and Flag-FAF1 was respectively induced by adding 100 ng/ml and 200 ng/ml tetracycline (Bioline, London, UK) for 24 hr.

For SILAC mass spectrometry, low passage U2OS cells were labelled using DMEM high-glucose media without arginine, lysine or methionine (Biosera, Boussens, France) reconstituted in filter-sterilized water supplemented with 3.7 g/l NaHCO_3_ and 10% dialyzed FBS (Biosera). The media was supplemented with 2 mM L-glutamine, 100 units/ml penicillin, 100 μg/ml streptomycin, 30 μg/ml methionine and respectively 146 μg/ml and 84 μg/ml of either lysine K0 and arginine R0 (Sigma) for light (K0R0) labelling or lysine K8 and arginine R10 (Cambridge Isotope Lab, Tewksbury, USA) for heavy (K8R10) labelling. After two weeks of culture in SILAC DMEM, the labelling efficiency was checked by mass spectrometry of random peptides.

Plasmid transfections were performed using TransIT-LT1 (Mirus, Madison, USA), following the manufacturer’s instructions. Briefly, approximately 3 × 10^6^ cells were cultured on a 15-cm plate. Then 24 hr later, the media was refreshed without antibiotics and the cells were transfected with 10 μg plasmid for 24 hr. siRNA transfections were performed for 48 hr, using oligos from Thermo Scientific at a final concentration of 5 nM and Lipofectamine RNAiMAX (Invitrogen) according to the manufacturer’s instructions. The following siRNA oligos were used: for FAF1 #0 (5′-CCACCUUCAUCAUCUAGUC-3′) [46] and siGENOME #1 (D-009106-01), #3 (D-009106-03), #4 (D-009106-04); for p97 (D-008727-06); for ASNA1 #3 (D-009666-03) and #18 (D-009666-18), and the Luciferase Duplex (P-002099) as non-targeting control.

### Cell extracts and immunoprecipitation

For immunoprecipitation, the cells were lysed in buffer A (50 mM N-2-hydroxyethylpiperazine-N'-2-ethanesulfonic acid (HEPES)/KOH, pH 7.2; 5 mM Mg(OAc)_2_; 70 mM KOAc; 0.2% Triton X-100; 10% glycerol; 0.2 mM ethylenediaminetetraacetic acid (EDTA); complete protease inhibitor cocktail (Roche, Mannheim, Germany). The lysates were incubated with anti-Flag beads (Anti-Flag M2 Affinity Gel, A2220, Sigma) or anti-VAPB (R2986, Division of Signal Transduction Therapy Unit (DSTT) at the University of Dundee) antibodies cross-linked to Protein A-Sepharose Fast Flow beads (PAS beads, GE Healthcare Life Sciences, Little Chalfont, UK). Uncoupled PAS beads were used as a control. Endogenous FAF1 immunoprecipitation was performed using anti-FAF1 antibodies (S370D, DSTT) and sheep IgG, without prior cross-linking to PAS beads. Immunoprecipitation was performed at 4°C for 2 hr with rotation. The beads were washed with buffer A and the proteins were eluted by incubating with 3× Laemmli buffer for 10 min in Micro Bio-Spin columns (BioRad, Hemel Hempstead, UK). Mouse brain tissue was homogenized in buffer A using a Polytron (Kinematica, Eschbach, Germany). The lysates were centrifuged for 10 min at 13,000 rpm, filtered using a 0.22 μm sieve and centrifuged again for 10 min at 13,000 rpm. The supernatants were pre-cleared by incubation with uncoupled PAS beads, three times for 20 min each, under rotation. All steps were done at 4°C. For mass spectroscopy, the immunoprecipitation was performed as described with the following changes: after washing with buffer A, the beads were additionally washed with 100 mM Tris-HCl pH 8.5 and the bound proteins were eluted by incubating with 8 M urea in 100 mM Tris-HCl pH 8.5 for 15 min at 37°C. The eluted proteins were reduced by incubation with 3 mM TCEP for 20 min and then with 11 mM iodoacetamide for 15 min, both at room temperature. Samples were pre-digested with 0.1 μg Lys-C (Roche) for 4 hr at 37°C, then diluted and the buffer adjusted so the final digestion buffer contained 2 M urea, 1 mM CaCl_2_ and 0.5 μg Trypsin (Roche). Trypsin digestion was performed at 37°C for 16 hr. The digested peptides were acidified to pH < 3 using 1% TFA, purified using C18 Silica microspin columns (The Nest Group, Southborough, USA), eluted in 0.1% TFA, 50% ACN and stored dry at -80°C until mass spectroscopy.

For SILAC immunoprecipitation, light- or heavy-labelled cells were treated with either MG132 or dimethyl sulfoxide as a control. The cells were independently lysed in buffer A and equal amounts of heavy and light lysate were mixed before incubation with the appropriate beads. The immunoprecipitation was performed as described above. For lysine-ϵ-GlyGly peptide purification, light-labelled cells were transfected with Flag-VAPB and heavy-labelled cells with Flag-FAF1, both treated with MG132 for 2 hr. The cells were independently lysed in buffer A, Flag-immunoprecipitated and trypsin digested as described above. The digested peptides were pooled together and the lysine-ϵ-GlyGly peptides were immunoaffinity purified using the PTMScan ubiquitin remnant motif (K-ϵ-GG) kit (Cell Signaling, Leiden, The Netherlands; 5562) as described by Udeshi *et al.*[37].

### Indirect immunofluorescence microscopy

The cells were plated on cover glass (thickness No 1.5) for 24 hr, followed by Flag-ASNA1 plasmid transfection or Flag-FAF1 tetracycline induction for 24 hr, and treated with MG132 for 2 hr where indicated. Cells were then fixed in 3.7% paraformaldehyde/PBS at room temperature for 15 min, permeabilized in 0.2% Triton X-100/PBS for 5 min, and blocked in 3% FBS in PBS/0.05% Tween for 45 min. Antibodies diluted in 3% FBS in PBS/0.05% Tween were sequentially added to the cells and incubated for 1 hr at room temperature followed by three washes with PBS/0.05% Tween. Cells were then incubated for 5 min with 4',6-diamidino-2-phenylindole (DAPI). Finally, the samples were washed three times with PBS/0.05% Tween, three times with PBS, and twice with water before mounting on microscope slides with Mowiol 4-88 (Polysciences, Eppelheim, Germany). Images were obtained with a DeltaVision Spectris microscope (Applied Precision, Issaquah, USA), using a CoolSNAP HQ camera (Roper Scientific, Martinsried, Germany) and a 100× 1.4 NA objective (Olympus, Southend-on-Sea, UK). The SoftWorx software (Applied Precision) was used for image acquisition and deconvolution.

### Recombinant protein expression and *in vitro* binding assays

GST-fusion proteins were expressed in BL21 cells at 15°C for 16 hr upon induction with 0.1 mM isopropyl β-D-1-thiogalactopyranoside. GST-TEV-VAPB proteins were captured on GSH-Sepharose, washed and then either eluted with 10 mM reduced glutathione or recovered by incubating the resin with TEV-His6 protease, which was subsequently removed by affinity chromatography. To remove the GST tag, GST-TEV-Flag-FAF1 was cleaved using AcTEV protease (Invitrogen) overnight at 4°C. Then 2 μg Flag-FAF1 was mixed with 0.5 μg of the indicated form of VAPB in a final volume of 400 μl buffer B (50 mM HEPES/KOH, pH 7.5; 60 mM KOAc; 5 mM MgCl_2_; 5% glycerol; 0.1% Triton X-100). BSA was added to control samples to reach the same final protein concentration. The protein mixtures were pre-incubated for 30 min at 4°C, then anti-Flag beads (Sigma) were added and incubated for another 30 min at 4°C, under rotation. The beads were washed and the bound proteins were eluted by incubation with 3× Laemmli buffer for 10 min at 70°C.

### Mass spectrometry analysis

The dried peptides were resuspended in 20 μl 0.1% (v/v) TFA and separated on a Dionex UltiMate 3000 LC system (Thermo Scientific) using a 25 cm column packed with 3 μm Magic C18 material (Michrom Bioresources, Auburn, USA). Mass spectra were acquired on an LTQ Orbitrap Velos mass spectrometer (Thermo Scientific) operating in data-dependent mode. After conversion to mzXML, the raw data were searched using Comet against version 3.87 of the IPI human or mouse protein database using static carboxamidomethylation of cysteine residues, variable oxidation of methionine residues and accounting for up to two missed tryptic cleavages. For SILAC samples, variable modification of lysine and arginine residues was also specified. The Trans-Proteomic Pipeline was used to assign peptide and protein probabilities and to filter results at a 1% false discovery rate. Data from the anti di-glycine remnant antibody analysis were searched using X!!TANDEM (v. 2010.12.01.1) with the k-score plugin. Two sets of static modifications were used: (1) carboxamidomethylation of cysteine and (2) carboxamidomethylation of cysteine, SILAC heavy Lys and Arg. Additionally, variable oxidation of methionine and GlyGly modification of lysine residues were accounted for.

### Antibodies and chemicals

The following antibodies were used: mouse anti-Flag M2 (Sigma, A8592), mouse anti-ubiquitin FK2 (Enzo, PW8810), rabbit anti-ubiquitin (Dako, Ely, UK; Z0458), mouse anti-p97 (Fitzgerald, North Acton, USA; 10R-P104A), rabbit anti-VAPA (Epitomics, Burlingame, USA; S1706), mouse anti-FAF1 (Abnova, Taipei City, Taiwan; H00011124-A01), rabbit anti-FAF1 (courtesy of Millipore, Billerica, USA), rabbit anti-GAPDH (Cell Signaling, 2118), rabbit anti-RAB3GAP1 (Proteintech, Manchester, UK; 21663-1-AP), rabbit anti-RAB3GAP2 (Abgent, Maidenhead, UK; AP9635B), rabbit anti-WDR44 (Bethyl, Montgomery, USA; A301-441A), mouse anti-ASNA1 (Abnova, H00000439-M03), rabbit anti-Syntaxin 1A (GeneTex, Irvine, USA; GTX113559), rabbit anti-BAG6 (Cell Signaling, 8523S), mouse anti-RPN2 (Abnova, H00006185-B01), rabbit anti-CD147 (Abcam, Cambridge, UK; ab108317), Alexa Fluor® 488 goat anti-rabbit (Invitrogen, A11008) and Alexa Fluor® 594 chicken anti-mouse (Invitrogen, A21201). Anti-VAPA (Epitomics, S1706) antibodies recognize both VAPA (upper band) and VAPB (lower band) in human cell extracts. The following antibodies were raised and affinity purified using the appropriate antigen by the DSTT: sheep anti-FAF1 (S370D; antigen human FAF1, 1-650), rabbit anti-VAPB (R2986 and R2987; antigen human VAPB, 1-210).

## Abbreviations

ALS: amyotrophic lateral sclerosis; BSA: bovine serum albumin; C-Ter: C-terminal half; DMEM: Dulbecco's modified Eagle's medium; DSTT: Division of Signal Transduction Therapy Unit; ER: endoplasmic reticulum; ERAD: endoplasmic-reticulum-associated degradation; FBS: fetal bovine serum; FFAT motif: two phenylalanines in an acidic tract motif; HEPES: N-2-hydroxyethylpiperazine-N'-2-ethanesulfonic acid; KM-DD: VAPB double mutant K87D M89D; MEM: modified Eagle's medium; MRC-PPU: Medical Research Council Protein Phosphorylation and Ubiquitylation Unit; OSBP: oxysterol-binding protein; PAS: Protein A-Sepharose; PBS: phosphate-buffered saline; PCR: polymerase chain reaction; RT-PCR: reverse-transcription-polymerase chain reaction; SILAC: stable isotope labeling by amino acids in cell culture; siRNA: small interfering RNA; TRC: transmembrane-domain recognition complex; UBA: ubiquitin associated; UBL: ubiquitin-like; VAP: vesicle-associated protein.

## Competing interests

The authors declare that they have no competing interests.

## Authors’ contributions

YB undertook all the experimental work presented in this study. PP and KT performed the mass spectrometry analysis. MW and NW generated the plasmid constructs. CJ and DF expressed and purified the recombinant proteins. YB and GA planned the experiments, analyzed the data and wrote the manuscript. All authors read and approved the final manuscript.

## Supplementary Material

Additional file 1: Table S1 Mass spectrometry analysis of Flag-FAF1 immunoprecipitates from human U2OS cells. Anti-Flag immunoprecipitates from untransfected cells were used as a negative control. Protein coverage and the share of spectrum IDs are indicated for each protein identified in the immunoprecipitates. Click here for file

Additional file 2: Figure S1 Ubiquitinated forms of VAPB are not detectable in Flag-VAPB immunoprecipitates. U2OS cells expressing Flag-VAPB from a tetracycline-inducible promoter were induced by addition of 100 ng/ml tetracycline. Flag-VAPB was immunoprecipitated after treatment with 10 μM MG132 for 1 or 2 hr, or 5 μM for 6 hr or from untreated cells (0 hr). Two exposures of Flag-VAPB immunoblots are shown. No ubiquitinated forms of Flag-VAPB can be detected with or without proteasome inhibition, not even after a very long exposure. Click here for file

Additional file 3: Table S2 Canonical FFAT proteins do not accumulate in endogenous VAPB immunoprecipitates upon proteasome inhibition. Click here for file

Additional file 4: Table S3 SILAC mass spectrometry analysis of endogenous VAPB immunoprecipitates from human U2OS cells. Light- or heavy-labeled cells were treated with MG132 for 2 or 6 hr, as indicated. Equal amounts of light- and heavy-labeled extracts were mixed and endogenous VAPB was immunoprecipitated using specific antibodies. The light/heavy SILAC ratios (L/H) determined by mass spectrometry are indicated, as well as the protein coverage. L + MG indicates that the light-labeled samples were treated with MG132. Proteins whose interaction with VAPB is stimulated by proteasome inhibition accumulate in these samples, resulting in L/H ratios higher than 1. H + MG indicates that the heavy-labeled samples were treated with MG132. Protein accumulation in the heavy-labeled samples results in L/H ratios lower than 1. The L/H for proteins that are not affected by proteasome inhibition will be close to 1. Click here for file

Additional file 5: Table S4 Mass spectrometry analysis of Flag-FAF1 immunoprecipitates from human U2OS cells treated with MG132 for 0, 2 or 6 hr as indicated. Anti-Flag immunoprecipitates from untransfected cells were used as a negative control. Protein coverage and the share of spectrum IDs are indicated for each protein identified in the immunoprecipitates. Click here for file

Additional file 6: Table S5 Ubiquitinated targets of VAPB and FAF1 identified by mass spectrometry upon enrichment of ubiquitinated peptides. A mixture of light-labeled Flag-VAPB and heavy-labeled Flag-FAF1 immunoprecipitates was analyzed by mass spectrometry after ubiquitinated peptide enrichment using antibodies specific to lysine-ϵ-GlyGly. The peptides containing lysine residues with an additional MW due to the GlyGly modification – 250.15 (heavy label) or 242.14 (light label) – are indicated for each protein. Click here for file

Additional file 7: Table S6 Mass spectrometry analysis of Flag-VAPA/B immunoprecipitates from human U2OS cells. Anti-Flag immunoprecipitates from untransfected cells were used as a negative control. Protein coverage and the share of spectrum IDs are indicated for each protein identified in the immunoprecipitates. Click here for file

Additional file 8: Table S7 Mass spectrometry analysis of endogenous VAPB immunoprecipitates from human HeLa cells. For the negative control sample, cell extracts were incubated with uncoupled Protein A-beads and the proteins retained on these beads were analyzed by mass spectrometry. Protein coverage and the share of spectrum IDs are indicated for each protein identified. Click here for file

Additional file 9: Table S8 Mass spectrometry analysis of endogenous VAPB immunoprecipitates from mouse brain. For the negative control sample, brain extracts were incubated with uncoupled Protein A-beads and the proteins retained on these beads were analyzed by mass spectrometry. Protein coverage and the share of spectrum IDs are indicated for each protein identified. Click here for file

Additional file 10: Figure S2 STX1A and B are not FFAT-like proteins. **(A)** Endogenous VAPB interacts with STX1A in mouse brain. **(B)** Alignment of the sequences that resemble FFAT motifs in human STX1A and B. **(C)** Flag-STX1A or B mutated for the two phenylalanine residues (F33A-F34A and F32A-F33A, respectively) in the putative FFAT motifs interact with VAPB similar to their WT counterparts. IP, immunoprecipitate; WT, wild type. Click here for file
