# Supplementary figures and images for: VAPB/ALS8 interacts with FFAT-like proteins including the p97 cofactor FAF1 and the ASNA1 ATPase
Source: BMC Biol. 2014 May 29;12:39. doi: 10.1186/1741-7007-12-39 (PMC4068158; doi:10.1186/1741-7007-12-39)

# Additional Figure 1

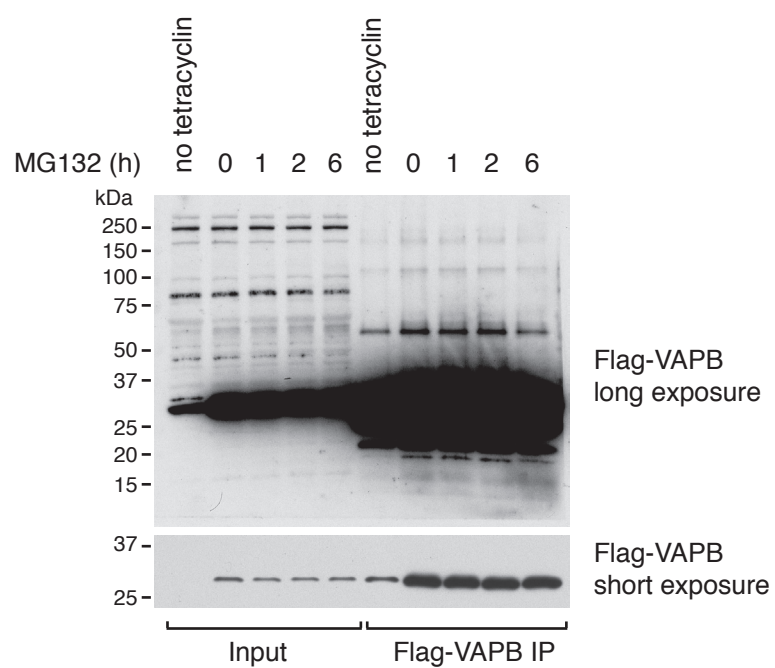

Supplement: Additional file 2: Figure S1 — Ubiquitinated forms of VAPB are not detectable in Flag-VAPB immunoprecipitates. U2OS cells expressing Flag-VAPB from a tetracycline-inducible promoter were induced by addition of 100 ng/ml tetracycline. Flag-VAPB was immunoprecipitated after treatment with 10 μM MG132 for 1 or 2 hr, or 5 μM for 6 hr or from untreated cells (0 hr). Two exposures of Flag-VAPB immunoblots are shown. No ubiquitinated forms of Flag-VAPB can be detected with or without proteasome inhibition, not even after a very long exposure. [file 1741-7007-12-39-S2.pdf]
