# Supplementary material for: VAPB/ALS8 interacts with FFAT-like proteins including the p97 cofactor FAF1 and the ASNA1 ATPase
Source: BMC Biol. 2014 May 29;12:39. doi: 10.1186/1741-7007-12-39 (PMC4068158; doi:10.1186/1741-7007-12-39)
Supplement: Additional file 3: Table S2 — Canonical FFAT proteins do not accumulate in endogenous VAPB immunoprecipitates upon proteasome inhibition. [file 1741-7007-12-39-S3.pdf]

Additional Table 2. Canonical FFAT proteins do not accumulate in endogenous VAPB immunoprecipitates upon proteasome inhibition

PROTEIN NAME	UniProt ID	MW (Da)	FFAT motif	SILAC ratio L/H[a]			
				L+MG 2h	H+MG 2h	L+MG 6h	H+MG 6h
**OSBP**	P22059	89421	**EFFDAPE**	1.08±0.17 (28)	1.18±0.12 (21)	1.28±0.17 (17)	1.09±0.10 (19)
**OSBP2**	Q969R2	101266	**EYFDAME**	-	0.95±0.02 (3)	-	-
**OSBPL2**	Q9H1P3	55201	**EFFDAVT**	1.01±0.24 (15)	1.30±0.31 (13)	0.97±0.16 (16)	1.12±0.20 (18)
**OSBPL3**	Q9H4L5	101224	**EFFDAQE**	1.02±0.08 (76)	1.06±0.12 (84)	1.15±0.14 (83)	1.03±0.09 (79)
**OSBPL6**	Q9BZF3	106306	**EFFDAQE**	1.13±0.07 (29)	0.89±0.10 (24)	1.11±0.15 (36)	1.03±0.07 (38)
**OSBPL9**	Q96SU4	83185	**EFYDADE**	0.99±0.07 (47)	-	-	-
**OSBPL10**[b]	Q9BXB5	83970	**EYYLTAF?**	0.95±0.11 (25)	0.89±0.09 (22)	1.02±0.12 (23)	1.23±0.15 (23)
**OSBPL11**[b]	Q9BXB4	83643	**EYYLTSF?**	1.04±0.06 (30)	1.09±0.10 (27)	1.07±0.15 (24)	1.22±0.11 (26)
**PITPNM1**	O00562	134848	**EFFDAHE**	1.20±0.07 (17)	1.02±0.05 (15)	1.20±0.06 (13)	1.08±0.11 (19)
RAB3GAP1[c]	Q15042	110524	EFFECLS	1.28±0.10 (20)	0.96±0.07 (16)	1.40±0.10 (31)	1.03±0.10 (25)
RAB3GAP2[c]	Q9H2M9	155985	NA	0.94±0.12 (6)	0.95±0.07 (4)	1.11±0.11 (10)	1.04±0.05 (13)

[a] Light or heavy labeled cells were treated with MG132 for 2 or 6 hours, as indicated. Equal amounts of light and heavy labeled extracts were mixed and endogenous VAPB was immunoprecipitated using specific antibodies. The light/heavy SILAC ratios (L/H) determined by mass spectrometry are indicated. L+MG indicates that the light-labeled samples were treated with MG132. Proteins whose interaction with VAPB is stimulated by proteasome inhibition accumulate in these samples, resulting in L/H ratios higher than one. H+MG indicates that the heavy-labeled samples were treated with MG132. Protein accumulation in the heavy-labeled samples results in L/H ratios lower than one. The L/H for proteins that are not affected by proteasome inhibition will be close to one. The numbers in parenthesis represent the number of spectra analyzed for each sample.

[b] OSBPL10 and 11 interact strongly with VAPB, although there is no obvious FFAT-like motif in their sequence.

[c] RAB3GAP1 and 2 behave like OSPBs in this analysis and they are included in the table, although the FFAT motif of RAB3GAP1 is non-canonical.
