# Supplementary material for: VAPB/ALS8 interacts with FFAT-like proteins including the p97 cofactor FAF1 and the ASNA1 ATPase
Source: BMC Biol. 2014 May 29;12:39. doi: 10.1186/1741-7007-12-39 (PMC4068158; doi:10.1186/1741-7007-12-39)

Additional Figure 2

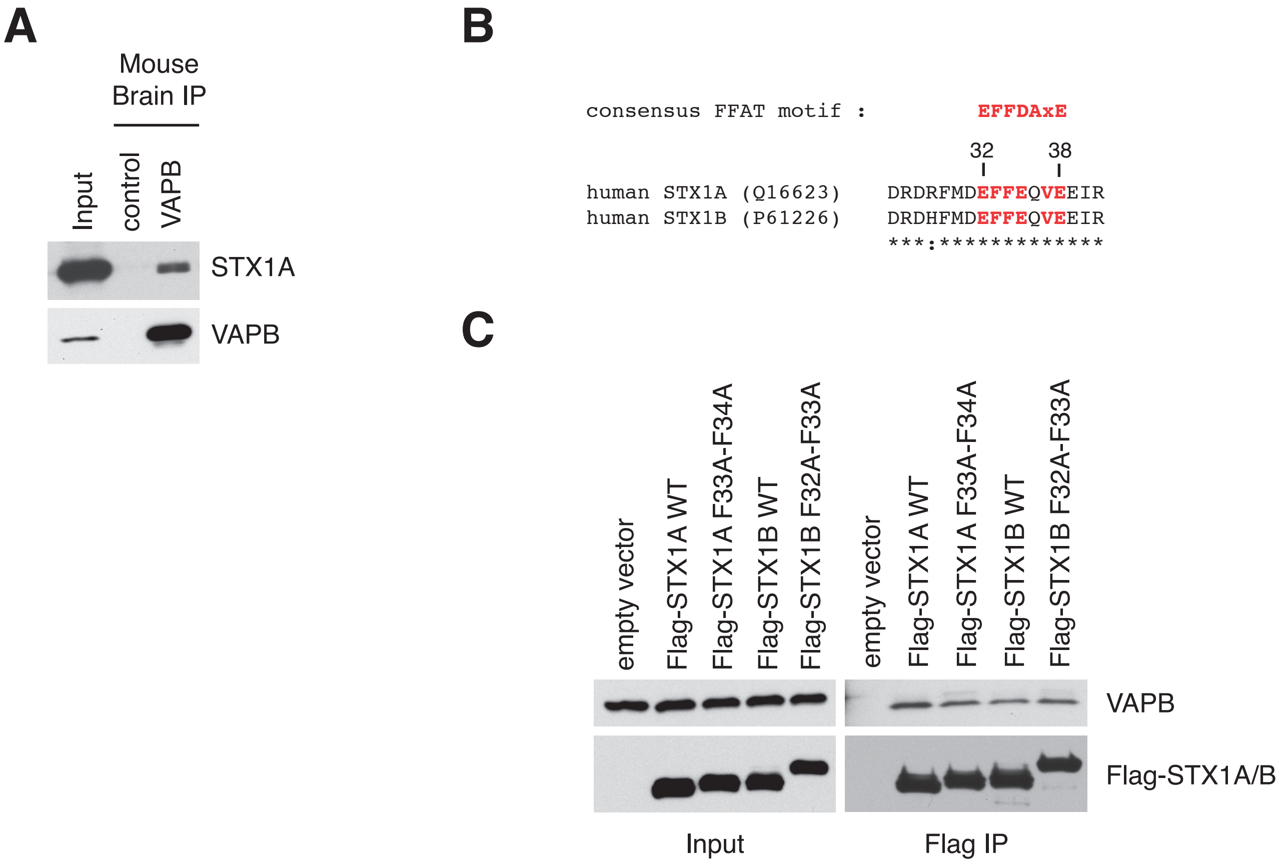

Supplement: Additional file 10: Figure S2 — STX1A and B are not FFAT-like proteins. (A) Endogenous VAPB interacts with STX1A in mouse brain. (B) Alignment of the sequences that resemble FFAT motifs in human STX1A and B. (C) Flag-STX1A or B mutated for the two phenylalanine residues (F33A-F34A and F32A-F33A, respectively) in the putative FFAT motifs interact with VAPB similar to their WT counterparts. IP, immunoprecipitate; WT, wild type. [file 1741-7007-12-39-S10.pdf]
